# Gestational weight gain and blood pressure control in physiological pregnancy and pregnancy complicated by hypertension

**DOI:** 10.34763/jmotherandchild.20222601.d-22-00020

**Published:** 2022-12-14

**Authors:** Tomasz Mikołaj Maciejewski, Ewa Szczerba, Agnieszka Zajkowska, Katarzyna Pankiewicz, Anna Bochowicz, Grzegorz Szewczyk, Grzegorz Opolski, Maciej Małecki, Anna Fijałkowska

**Affiliations:** Department of Obstetrics and Gynecology, Institute of Mother and Child, Warsaw, Poland; Department of Cardiology, Institute of Mother and Child, Warsaw, Poland; First Chair and Department of Cardiology, Medical University of Warsaw, Warsaw, Poland; Department of Applied Pharmacy, Medical University of Warsaw, Warsaw, Poland; Department of Biophysics, Physiology and Pathophysiology, Medical University of Warsaw, Warsaw, Poland; Department of Obstetrics, Perinatology and Gynecology, Medical University of Warsaw, Warsaw, Poland

**Keywords:** Gestational hypertension, chronic hypertension in pregnancy, gestational weight gain, blood pressure control, hypertensive disorders in pregnancy

## Abstract

**Background:**

Obesity is a widely recognised risk factor for chronic and gestational hypertension. Influence of gestational weight gain on blood pressure control throughout the pregnancy is not well characterised.

**Material and methods:**

Women in the third trimester of a singleton pregnancy were recruited to the study. Medical records were analysed and a special survey was conducted to obtain history on hypertensive disorders in pregnancy and weight changes during pregnancy. Blood pressure measurements were taken during the office visit in line with international guidelines. Relationships between gestational weight gain and maximal and office values of systolic and diastolic blood pressure values were analysed.

**Results:**

Data of 90 women in normal pregnancy, 40 with gestational hypertension and 21 with chronic hypertension were analysed. Gestational weight gain was 11.9 ± 4.6 kg in the normal pregnancy group, 13.0 ± 5 kg in the gestational hypertension group and 10.6 ± 3.4 kg in the chronic hypertension group. Gestational weight gain positively correlated with both office (r = 0.48; p < 0.001) and maximal blood pressure values (r = 0.34; p = 0.004) in normal pregnancy and with maximal blood pressure values (r = 0.57; p = 0.02) in women with chronic hypertension. No correlation was observed between gestational weight gain and blood pressure values among women with gestational hypertension.

**Conclusion:**

In normal pregnancy and in women with chronic hypertension greater gestational weight gain is related to higher blood pressure values in the third trimester.

## Introduction

Hypertensive disorders in pregnancy (HDP) including pre-existing/chronic hypertension (CH), gestational hypertension (GH), pre-existing hypertension with superimposed gestational hypertension with proteinuria and antenatally unclassifiable hypertension are associated with different risk factors and complication rates in pregnancy [1]. Although the rate of GH in Europe and the USA is declining slightly, it still affects 4–8% of pregnancies [2]. Understanding the pathophysiology and identifying risk factors, especially modifiable ones, for rises in blood pressure during pregnancy is essential in prevention of some types of HDP and minimisation of complication rates [3].

With every 1 kg/m^2^ of prepregnancy body mass index (BMI), the risk for GH rises by 6% [4]. Gestational hypertension increases the risk for future hypertension, coronary heart disease, and stroke [5]. Overweight and obesity are one of the modifiable risk factors for HDP [6].

There is emerging evidence on the role of gestational weight gain (GWG) on short- and long-term maternal and neonatal health outcomes. Additionally, GWG has been linked with increased risk of obesity in the mother [7] and cardio-metabolic outcomes in children [8].

During normal pregnancy blood pressure decreases in the first and second trimester and begins to rise from the mid-third trimester, achieving prepregnancy values [9]. The factors influencing blood pressure profile throughout the pregnancy are not fully described. There seems to be a complex relationship between prepregnancy weight and BMI, gestational weight gain and blood pressure profile during pregnancy and in later life. So far, few studies have assessed the relationship between GWG and blood pressure profile in pregnant women. There is little data on factors influencing blood pressure control in CH women during pregnancy. Women with CH were often excluded from the analysis [10]. Data comparing GWG in different subpopulations of women with HDP and its impact on blood pressure control during pregnancy is lacking. The question whether pregnancy blood pressure profile itself could also provide information on stratification of cardiovascular risk remains to be uncovered and therefore it is important to identify factors influencing changes in blood pressure during pregnancy. We aimed at describing the effects of GWG on blood pressure values during the third trimester of pregnancy in GH and CH as well as in normal pregnancy. Our hypothesis was that GWG will have different impact on blood pressure control in those groups.

## Material and methods

### Study population and protocol

Women in the third trimester of a singleton pregnancy were recruited for the study. This was a single-center prospective trial carried out between October 2014 and June 2017. Data regarding anthropometric and demographic factors – such as height, pregestational weight, gestational weight at the time of the examination, the history of hypertensive disease – were obtained by a questionnaire and compared, when available, with obstetric records to ensure accuracy. The analysed information regarding hypertension includes the time of diagnosis, highest values of systolic and diastolic blood pressure during pregnancy, treatment, and number of ambulatory blood pressure measurement tests. *Chronic hypertension* was defined as hypertension diagnosed before or during the first 20 weeks of pregnancy. Women with secondary hypertension were excluded from the study. Hypertension diagnosed after the twentieth week of pregnancy constituted *gestational hypertension*. As no data on postpartum resolution of blood pressure abnormalities were collected, this criterion was not a part of the used definition of gestational hypertension. There were no women with pre-existing hypertension with superimposed gestational hypertension with proteinuria in the studied group. This is a substudy of a project financed by the National Science Centre, Kraków, Poland (NCN 2013/11/N/NZ5/03388). The project was approved by the local Ethical Committee (opinion number 17/2013). Women were included in the study after providing a written informed consent.

### Blood pressure measurement

Each women had an office blood pressure measurement obtained according to recommended guidelines with an Omron device certified for use during pregnancy [11]. Measurements were obtained on both arms.

### Statistical analysis

We analysed the results for women in normal pregnancy and for women with hypertensive disorders of pregnancy separately. Afterwards a subanalysis of women with CH and GH was carried out. Body mass index (BMI) was calculated as weight in kilograms / height in meters squared. Descriptive data are presented as a mean or median depending on the distribution pattern. Differences between the groups regarding weight and BMI were calculated by Mann–Whitney U test. We decided to use the nonparametric test because of the size of the compared groups. Spearman correlation was calculated to examine the relationship between anthropometric parameters and blood pressure in each prespecified subgroup. SPSS Statistics 23 statistical software was used for the analysis. P value of less than 0.05 was considered as statistically significant.

## Results

### Study population characteristics

The study group consisted of 61 women with HDP, 40 with GH, and 21 with CH, and 90 women in normal pregnancy. They were assessed during the third trimester. Between women in normal pregnancy and HDP there were no differences in age (30.5 ± 4.1 vs. 32 ± 4.7 years; p = 0.14) nor duration of pregnancy (32 ± 3.4 vs. 34 ± 3.8 weeks; p = 0.16). Subanalysis of the HDP group showed that women with GH had slightly higher gestational age compared with CH group at the time of assessment (Table 1). In the normal pregnancy group, 10 women (11%) were overweight and 5 (6%) were obese before pregnancy. In comparison 37.5% (15/40) of the GH and 38.1% (8/21) of the CH group were overweight before pregnancy. Obesity appeared in 20% (8/40) of GH group and 4.8% (1/21) of CH group. In total, prepregnancy weight and BMI were higher in the HDP group than in women with normal pregnancy (for weight 63.7 vs 72.1 kg; p < 0.001; for BMI 22.8 vs 26.2 kg/m2; p < 0.001). Interestingly, the change in weight (11.9 ± 4.6 vs. 12.2 ± 4.2 kg; p = 0.512) and in BMI (4.2 ± 1.8 vs. 4.4 ± 1.9 kg/m2; p = 0.352) during pregnancy were not different between the groups.

**Table 1:** Comparison of anthropometric and blood pressure measurement data with division between three studied groups. DBP – diastolic blood pressure; HR – heart rate; SBP – systolic blood pressure.

	**normal pregnancy**	**hypertensive disorders in pregnancy**	**p**	**gestational hypertension**	**chronic hypertension**	**p**
prepregnancy weight [kg]	**63.7**	**72.1**	**<0.001**	74	70	0.288
prepregnancy body mass index [kg/m2]	**22.8**	**26.2**	**<0.001**	26.1	25.8	0.532
weight change [kg]	11.9	12.2	0.512	13	10.8	0.163
body mass index change [kg/m2]	4.2	4.4	0.352	4.8	3.7	0.280
age [years]	30.5	32	0.14	31.5	33	0.212
pregnancy duration	32	34	0.16	**35**	**33**	**0.04**
office left arm SBP [mmHg]	**115**	**134**	**<0.001**	**138**	**129**	**0.018**
office left arm SBP [mmHg]	**70**	**85**	**<0.001**	**87.5**	**80**	**0.034**
office right arm SBP [mmHg]	**117**	**132**	**<0.001**	**136**	**128.8**	**0.015**
office right arm SBP [mmHg]	**70**	**85.5**	**<0.001**	**87**	**78.5**	**0.008**
maximal SPB [mmHg]	**130**	**160**	**<0.001**	160	155	0.247
maximal DBP [mmHg]	**80**	**100**	**<0.001**	99.5	100	0.579
HR [beats/minute]	86	90.5	0.3	88	92.5	0.794

### Blood pressure measurement results

Both office systolic and diastolic blood pressure differed significantly among all three studied groups. Maximal blood pressure values were similar between the GH and CH group. Details are provided in Table 1.

### Correlations between blood pressure and weight: normal pregnancy and hypertensive disorders in pregnancy

**Graph 1:**
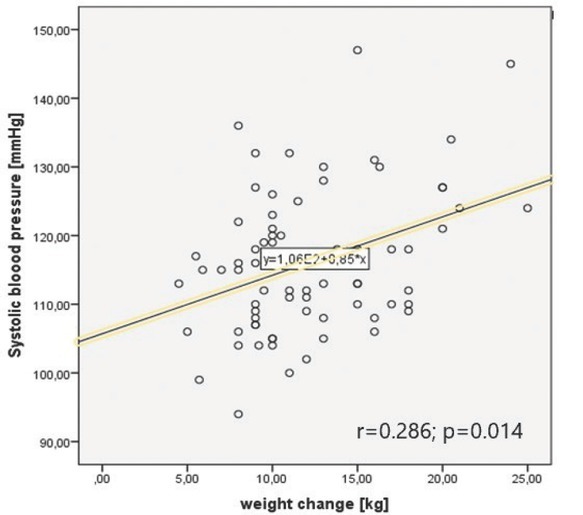
Correlation between systolic blood pressure and weight change in normal pregnancy group.

**Graph 2:**
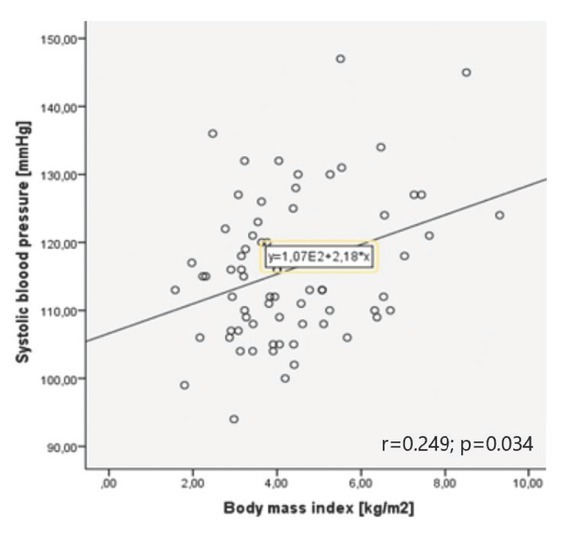
Correlation between systolic blood pressure and body mass index change in normal pregnancy group.

In the normal pregnancy group, higher GWG was associated with higher office and maximal measurements of blood pressure (Table 2, Graphs 1 and 2).

**Table 2:** Correlations between gestational weight gain and blood pressure control in the studied subgroups. DBP – diastolic blood pressure; HR – heart rate; SBP – systolic blood pressure.

	**normal**	**hypertensive disorders**	**gestational**	**chronic**
	**pregnancy**	**in pregnancy**	**hypertension**	**hypertension**
	**r = 0.286**	r = 0.233	r = 0.264	r = 0.022
office left arm SBP [mmHg] and gestational weight gain	**p = 0.014**	p = 0.115	p = 0.131	p = 0.943
office left arm DBP [mmHg] and gestational weight gain	r = 0.221	r = 0.25	r = 0.147	r = 0.329
	p = 0.06	p = 0.09	p = 0.407	p = 0.272
	**r = 0.479**	**r = 0.379**	**r = 0.338**	r = 0.408
office right arm SBP [mmHg] and gestational weight gain	**p < 0.001**	**p = 0.008**	**p = 0.05**	p = 0.147
	**r = 0.333**	r = 0.174	r = 0.085	r = 0.308
office right arm DBP [mmHg] and gestational weight gain	**p = 0.005**	p = 0.237	p = 0.632	p = 0.285
maximal SPB [mmHg] and gestational weight gain	**r = 0.34**	r = 0.165	r = -0.097	**r = 0.572**
	**p = 0.004**	p = 0.253	p = 0.584	**p = 0.021**
	**r = 0.37**	r = 0.063	r = -0.252	**r = 0.739**
maximal DBP [mmHg] and gestational weight gain	**p = 0.002**	p = 0.665	p = 0.15	**p = 0.001**
	**r = 0.24**	r = 0.148	r = 0.182	r = 0.079
HR [beats/minute] and gestational weight gain	**p = 0.038**	p = 0.315	p = 0.302	p = 0.788

Cumulative analysis of HDP group showed no correlation between blood pressure control parameters and GWG and BMI gain nor with prepregnancy weight and BMI values (Table 2).

**Graph 3:**
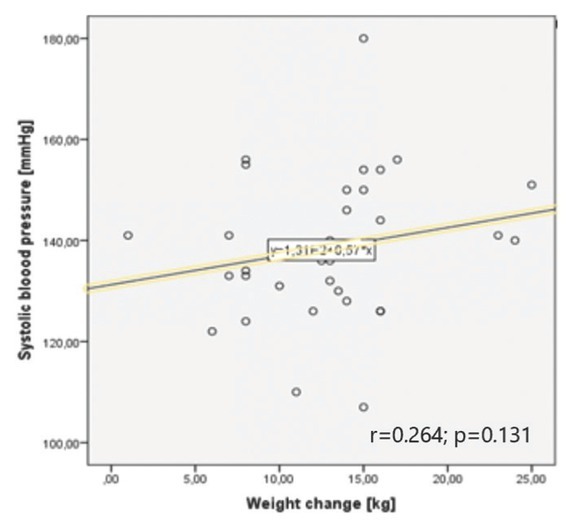
Correlation between maximal systolic blood pressure and weight change in gestational hypertension group.

**Graph 4:**
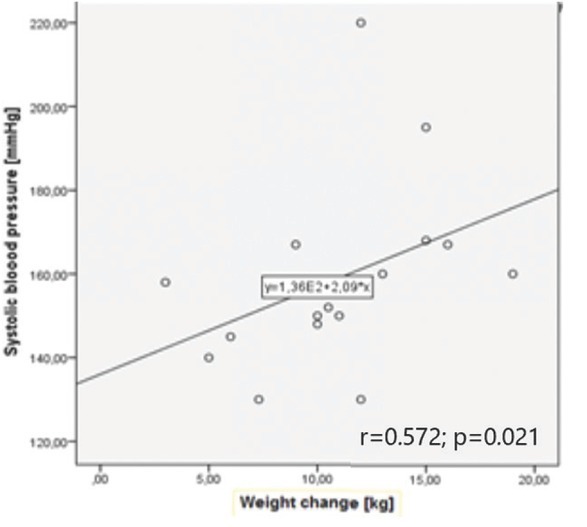
Correlation between maximal systolic blood pressure and weight change in chronic hypertension group.

**Correlations between blood pressure and weight: gestational and chronic hypertension group subanalysis** Correlation analysis between blood pressure measurements and anthropometric parameters was calculated in both types of HDP. Interestingly, although there were no correlations present in the GH group, a correlation between GWG and both maximal systolic and maximal diastolic blood pressure values measured during pregnancy were found in the CH group. A similar correlation with BMI change was noticed (for mSBP r = 0.561; p = 0.029 and for mDBP r = 0.681; p = 0.005). Details are shown in Table 2 and Graphs 3 and 4.

## Discussion

The present study is the first to demonstrate the different impact on blood pressure control of gestational weight gain in women with GH and CH. Additionally we observed that the higher the GWG is, the greater the blood pressure values are during normal pregnancy.

Excess GWG is a major health concern affecting almost half of pregnant women [12]. So far GWG has been described as a risk factor for GH and for superimposed preeclampsia but not as a risk factor for poorer control of blood pressure during pregnancy in patients with chronic hypertension. Most of the available data are retrospective and analyse the relationship between GWG and final diagnosis of GH, not with the values of blood pressure, as will be shown further in the discussion.

### Gestational weight gain modifies blood pressure profile in pregnancy

Presented results show that greater GWG up to the third trimester of pregnancy correlates with higher office and maximal blood pressure measurements. This finding is in line with evidence from the literature. In a study of 158 healthy pregnant women, GWG in the second and third tertiles resulted in lack of mid-trimester drop in systolic and diastolic blood pressure [13]. Macdonald-Wallis et al. describe results of 12,522 women from the Avon Longitudinal Study of Parents and Children [10]. They found that in normotensive pregnant women, with every 200g of gestational weight gain to 18 weeks of pregnancy there is about 30% of increase in risk of GH and preeclampsia, irrespective of prepregnancy weight. Lei et al. analysed the relation between trajectories of diastolic blood pressure values and GWG and found that women with highly increasing weight gain trajectory were at greater risk of being in the high-J shaped trajectory for diastolic pressure values [14]. This subgroup also had the highest values of systolic blood pressure values throughout the pregnancy. It seems also that prepregnancy weight gain leads to an increased risk of HDP [15].

Despite those findings there are no clinical recommendations regarding more screening, for example with ambulatory blood pressure monitoring, in women with excessive gestational weight gain for early diagnosis of HDP.

### Gestational weight gain as a risk for GH and GH control

Excessive gestational weight gain, especially in early pregnancy, is recognised as a risk factor for GH, even independently of obesity prior to pregnancy [16]. Additionally, the greater GWG the higher this risk is across different races [17,18]. There are no data on the value of GWG and the control of GH once the diagnosis is made.

### Gestational weight gain as a risk factor for poorer control of CH during pregnancy

In our study there was a positive correlation between GWG and maximal systolic and diastolic blood pressure values during pregnancy, suggesting that it might be one of the parameters influencing blood pressure control. We did not observe correlation between GWG and measurement results obtained in the office, probably as a result of proper hypotensive treatment throughout further pregnancy.

No data on influence of GWG on blood pressure profile control in pregnant women with chronic hypertension were found. However, it is known that higher than recommended GWG increases maternal, obstetric and neonatal risks in this subgroup [19]. Excessive GWG in women without prepregnancy overweight or obesity aggravates risk of superimposed preeclampsia 3.5 times [20].

## Conclusion

In the third trimester of a normal pregnancy, a greater GWG is related to higher blood pressure values. Despite that in women with hypertensive disorders in pregnancy such dependency was not observed, a separate analysis revealed that greater weight gain in women with CH is related to higher maximal values of systolic and diastolic blood pressure. These observations underline pathophysiological differences between CH and GH. The GWG has similar influence on blood pressure in normal pregnancy and in women with CH during pregnancy, but not in women with a diagnosis of GH. This study highlights the need for further assessment of GWG and blood pressure profiles in pregnancy.

### Limitations

There are several limitations of our study. First, a small number of cases for particular subtypes of the HDP group was included. Second, we did not analyse the influence of GWG on pregnancy complications in the studied patients. Third, a 24-h ambulatory blood pressure monitoring was not performed as a part of the study protocol, which would have provided more averaged values of the measurements. And finally, the impact of treatment with antihypertensive medications was not evaluated.

## ORCID IDs of the Authors:

Tomasz Mikołaj Maciejewski 0000-0003-3761-1924 (https:// orcid.org/0000-0003-3761-1924)

Ewa Szczerba 0000-0002-4921-4726 (https://orcid.org/0000-0002-4921-4726)

Agnieszka Zajkowska 0000-0002-6664-9705 (https://orcid.org/0000-0002-6664-9705)

Katarzyna Pankiewicz 0000-0001-7756-1963 (https://orcid.org/0000-0001-7756-1963)

Anna Bochowicz^†^ 0000-0002-8773-0908 (https://orcid.org/0000-0002-8773-0908)

Grzegorz Szewczyk 0000-0003-4143-2777 (https://orcid.org/0000-0003-4143-2777)

Grzegorz Opolski 0000-0003-4744-2554 (https://orcid.org/0000-0003-4744-2554)

Maciej Małecki 0000-0002-7078-4918 (https://orcid.org/0000-0002-7078-4918)

Anna Fijałkowska 0000-0002-2225-9684 (https://orcid.org/0000-0002-2225-9684)

## Contributorship

Tomasz Mikołaj Maciejewski: Conceptualisation, methodology, investigation, data curation, supervision, project administration, visualisation, writing – original draft, writing – review & editing

Ewa Szczerba: Conceptualisation, methodology, investigation, resources, data curation, formal analysis, visualisation, funding acquisition, writing – original draft, writing – review & editing

Agnieszka Zajkowska: Conceptualisation, investigation, resources, data curation, formal analysis, writing – review & editing

Katarzyna Pankiewicz: Data curation, investigation, resources, writing – review & editing

Anna Bochowicz: Data curation, methodology, investigation, resources, supervision

Grzegorz Szewczyk: Data curation, investigation, resources, supervision, writing – review & editing

Grzegorz Opolski: Conceptualisation, methodology, supervision, writing – review & editing

Maciej Małecki: Conceptualisation, methodology, project administration, supervision, writing – review & editing

Anna Fijałkowska: Conceptualisation, methodology, investigation, resources, data curation, formal analysis, visualisation, supervision, project administration, funding acquisition, writing – review & editing
